# A PLGA/Silk Fibroin Nanofibre Membrane Loaded with Natural Flavonoid Compounds Extracted from Green Cocoons for Wound Healing

**DOI:** 10.3390/ijms25179263

**Published:** 2024-08-27

**Authors:** Xiang Chen, Jiaqi Liu, Yaru Lu, Huijun Liu, Lan Cheng, Zhi Li, Fangyin Dai

**Affiliations:** 1 State Key Laboratory of Resource Insects, Institute of Sericulture and Systems Biology, Southwest University, Chongqing 400715, China; chenxiang262@163.com (X.C.);; 2 Chongqing Engineering Research Center of Biomaterial Fiber and Modern Textile, College of Sericulture, Textile and Biomass Science, Southwest University, Chongqing 400715, China; 3 Key Laboratory of Sericulture Biology and Genetic Breeding, Ministry of Agriculture and Rural Affairs, College of Sericulture, Textile and Biomass Sciences, Southwest University, Chongqing 400715, China

**Keywords:** green cocoons, flavonoids, electrospinning, silk fibroin, wound healing

## Abstract

The use of biodegradable materials combined with natural metabolites in wound dressings has received much attention. Flavonoids (FLs) from green cocoons, as metabolites, have antibacterial, antioxidant, anti-inflammatory, and other pharmacological effects. In this study, composite membranes of FL-loaded polylactic glycolic acid (PLGA)/silk fibroin (SF) were prepared by an electrospinning method. The prepared membranes, including SF, exhibited a good slow-release effect and cytocompatibility. An in vitro evaluation of the FL-loaded PLGA/SF membranes demonstrated good antioxidant, antibacterial, and anti-inflammatory properties. Animal experiments showed that the wound healing rate of PLGA/SF-2.5FL membranes within 15 days was 97.3%, and that of the control group was 72.5%. The PLGA/SF-2.5FL membranes shortened the inflammatory period of a full-layer wound model and promoted skin regeneration and wound healing by downregulating expression of the pro-inflammatory cytokines IL-1β and TNF-α and promoting expression of the growth factors VEGF, TGF-β, and EGF. In summary, the PLGA/SF-2.5FL composite nanofibre membrane with anti-inflammatory properties is an ideal wound dressing to promote acute wound healing.

## 1. Introduction

The skin is the largest organ of the human body, accounting for approximately 15% of the body’s total weight, and its main functions are preventing an organism from drying out and protecting the internal structure from the environment [1]. Structural damage and dysfunction of healthy skin is caused by trauma, genetic disease, acute trauma, heat injury, and even surgical procedures [2]. From a clinical point of view, wounds can be classified as either acute or chronic [3]. Acute wounds can heal spontaneously within 8–12 weeks, whereas chronic wounds usually take longer to heal and can be caused by a variety of factors including tumours, infections, or physical factors [3,4]. Skin wound repair is a dynamic process that is achieved through four stages: haemostasis, inflammation, proliferation, and tissue remodelling [5,6]. Considering the complexity of the healing mechanism and the multiple factors involved, selecting the appropriate wound dressing is crucial. The ideal wound dressing should expedite wound healing, minimize patient discomfort, facilitate the removal of excessive exudates, enhance autolysis debridement, keep the wound moist, reduce the wound temperature, and promote blood flow to achieve wound healing [7]. In addition, wound dressings should be antibacterial, non-toxic, elastic, viscous, and easy to disassemble [8,9].

Electrospun nanofibres can improve the repair or regeneration of various tissues, including nerve injury repair, myocardial defect repair, blood vessel bridging, wound healing, and interface construction between different tissues [10,11,12,13]. Electrospun membranes have many advantages for drug and molecular loading applications, such as the ability to encapsulate or load multiple drugs, good bioavailability, controlled release or dissolution, and biodegradability [14]. Therefore, electrospun membranes are an ideal choice for wound dressings because of their ability to regulate the behaviour of cells for regeneration, repair, and proliferation of tissues [15]. A poly(lactic-co-glycolic acid) (PLGA)/silk fibroin (SF)/artemisinin electrostatic spinning composite membrane prepared by Peng et al. [16] has anti-inflammatory activity and sustained slow-release of drugs, which further promotes wound healing. The fibre membrane prepared by electrospinning technology has excellent properties, such as interconnected voids, a high specific surface area, excellent mechanical properties, and a structural morphology similar to the extracellular matrix, all of which are more conducive to cell adhesion and proliferation [17]. In addition, PLGA has become the most commonly used electrospinning material due to its good biocompatibility, controllable drug release, and adjustable degradation rate, and is widely used in the field of nerve, skin, muscle, and bone tissue engineering [18,19,20].

Flavonoids are present in the cocoons of different silkworm species, with yellow-green cocoons containing mostly flavonoids. These flavonoids are mainly found in the sericin layer of silk. However, during the processing of the silk, most of the flavonoids are lost during the removal of sericin, causing the flavonoids to be wasted [21,22]. Flavonoids and flavonoid glycosides are natural products that exhibit strong antioxidant, antibacterial, and anti-inflammatory activity. The antioxidant properties of flavonoids are primarily due to their ability to react with and stabilize free radicals, as well as reduce their generation and cellular damage; their antibacterial effects destroy the normal function of bacteria by interfering with the metabolic processes of bacteria and the synthesis of the cell wall [23,24]. Wang et al. demonstrated that flavonoids extracted from yellow-green cocoons exhibited anti-fibrotic and anti-inflammatory activities, reduced oxidative stress, and had a protective role in the kidney [25]. Flavonoids, such as rutin, have been reported to prevent extracellular matrix accumulation and inhibit oxidative stress in the kidneys of rats with hyperglycaemic nephropathy, as well as having anti-inflammatory, antibacterial, antioxidant, and anti-ultraviolet properties [26,27]. Natural medical textile materials such as silk fibroin (SF), collagen fibre, and chitosan fibre can also be used to prepare tissue-engineered scaffolds. Among these materials, SF is noted for its excellent biocompatibility, controllable degradation, positive effect on wound healing, and interaction with fibrin and platelets to achieve haemostasis [28,29]. Studies have shown that implantation of SF induces virtually no immune response, as its implantation can be degraded by controlled protease-mediated digestion and can be combined with functional groups or RGD peptide modifications to promote cell adhesion [30].

In this study, electrospun nanofibre films were prepared using flavonoids (FLs) and SF of green cocoons (from the silkworm strain G200) as raw materials. By adding PLGA to increase the mechanical properties of the nanofibre membrane, FLs were loaded on the electrospun nanofibre membrane as a bacteriostatic and anti-inflammatory agent. The morphology, physicochemical properties, and slow-release properties of the prepared PLGA/SF-nFLs membranes were evaluated. Furthermore, the biological properties of these electrospun nanofibre dressings were assessed through in vitro testing for biocompatibility and anti-inflammatory effects, and through in vivo testing for wound closure, histopathology, and relative gene expression.

## 2. Results and Discussion

### 2.1. Characterization of PLGA/SF Nanofibre Membranes

**Figure 1:**
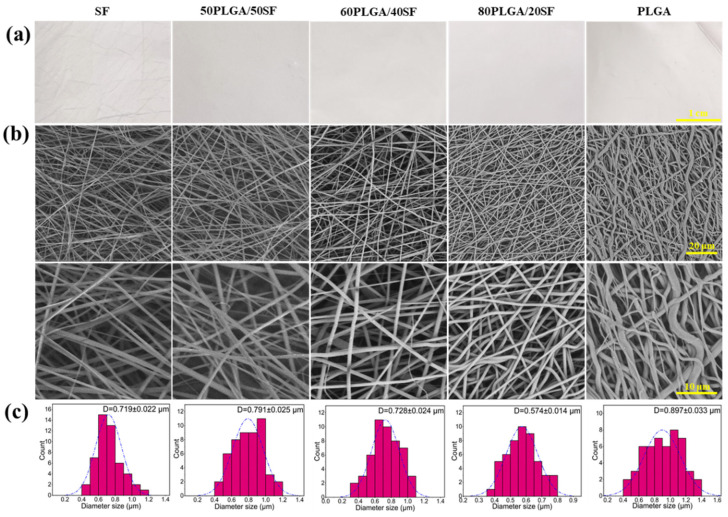
The optical photographs (**a**), SEM images (**b**), and the fibre diameter distributions (**c**) of the composite membranes.

Five types of nanofibre membranes were fabricated using electrospinning, SF, 50PLGA/50SF, 60PLGA/40SF, 80PLGA/20SF, and PLGA. The apparent morphology of these nanofibre films was observed by SEM. Figure 1 displays the resulting images, which show that all samples had smooth surfaces with a uniform fibre structure and no beaded string morphology. The average diameters of SF, 50PLGA/50SF, 60PLGA/40SF, 80PLGA/20SF, and PLGA were 719 ± 22, 791 ± 25, 728 ± 24, 574 ± 14, and 897 ± 33 nm, respectively. The statistical results showed that the addition of SF significantly reduced the average diameter of the nanofibre membranes. This was mainly because SF is an amphiphilic polymer electrolyte with hydrophobic and hydrophilic properties, and the addition of SF to the spinning solution led to an increase in charge density and conductivity. This enhanced the tensile effect of static electricity on the jet, resulting in a reduction in the diameter of the fibre [31].

**Figure 2:**
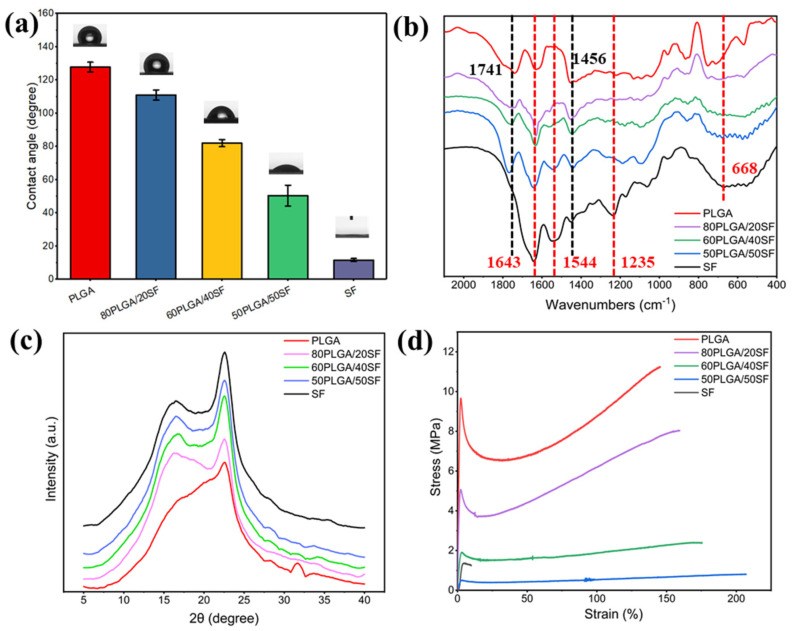
WCA (**a**), FTIR spectra (**b**), XRD spectra (**c**), and stress−strain curves (**d**) of different membranes.

The hydrophilicity of biomaterials is a crucial property of wound dressings, as it promotes the adhesion, growth, migration, and differentiation of cells. As shown in Figure 2a, the WCA of pure PLGA and pure SF was 127.8° and 11.6°, respectively. The WCA of the PLGA nanofibre membrane gradually decreased with the addition of SF, indicating an improvement in the hydrophilicity of the nanofibre membrane, which is beneficial to cell growth. Figure 2b shows the results of the structural conformation analysis of the electrospun nanofibre membranes using FTIR. The FTIR spectrum of pure PLGA showed characteristic absorption peaks at 1741 and 1456 cm^−1^ for C=O stretching and methyl stretching, respectively. The characteristic absorption peaks of amide I-IV of SF can be observed at 1643, 1544, 1235, and 668 cm^−1^, respectively. The characteristic absorption peaks of PLGA and SF were found in the FTIR spectra of nanofibre films with different proportions of PLGA and SF. The characteristic peaks of PLGA and SF did not shift or disappear significantly, suggesting the absence of any significant chemical interaction between PLGA/SF nanofibre membranes.

The crystallization process of the electrospun nanofibre films was characterized by XRD. The XRD patterns (Figure 2c) showed that pure SF had obvious diffraction peaks at 2θ = 16.7° and 22.6°, corresponding to *β*-sheet and α-helical conformations, respectively. PLGA/SF nanofibre membranes with different compositions also exhibited characteristic peaks at these angles, indicating that the SF structure conformation in the nanofibre membranes was unaffected by PLGA. The intensity of the characteristic peak was related to the SF content. To ensure that the nanofibre membranes prepared for wound healing had mechanical properties similar to skin tissue, their mechanical properties were measured, the results of which are shown in Figure 2d. The fracture strength of the samples ranged from 0.8 ± 0.1 to 11.2 ± 0.3 MPa, while the elongation at break ranged from 9.9 ± 0.2% to 207.1 ± 2.6%. The stress of the prepared nanofibre membranes fell within the stress range of human skin, ranging from 2.5 to 16.0 MPa [32,33]. SF blending can significantly increase the elongation at break of the nanofibre film and reduce the breaking strength, but it can also meet the needs of skin wound healing. Therefore, to utilize electrospun nanofibre membranes as a wound dressing for skin wound healing, 80PLGA/20SF (P-SF) was used for subsequent experiments.

### 2.2. Fabrication and Characterization of FL-Loaded P-SF Nanofibre Membranes

Using P-SF as a substrate, five types of membranes, P-SF, P-SF-0.5FLs, P-SF-1.0FLs, P-SF-2.5FLs, and P-SF-5.0FLs, were prepared by electrospinning with different concentrations of FLs (Figure S1a). The SEM of the nanofibre membranes is shown in Figure S1b, and it can be observed that the addition of FLs did not affect the process of spinning the fibres. A statistical analysis of the fibre diameters determined by SEM showed that with the addition of FLs, the diameters of the composite nanofibre films from a low concentration to a high concentration were 607 ± 16, 519 ± 26, 369 ± 32, and 318 ± 14 nm (Figure S1c). It can be seen from these results that the diameter of the fibres gradually decreased as the FL concentration increased.

**Figure 3:**
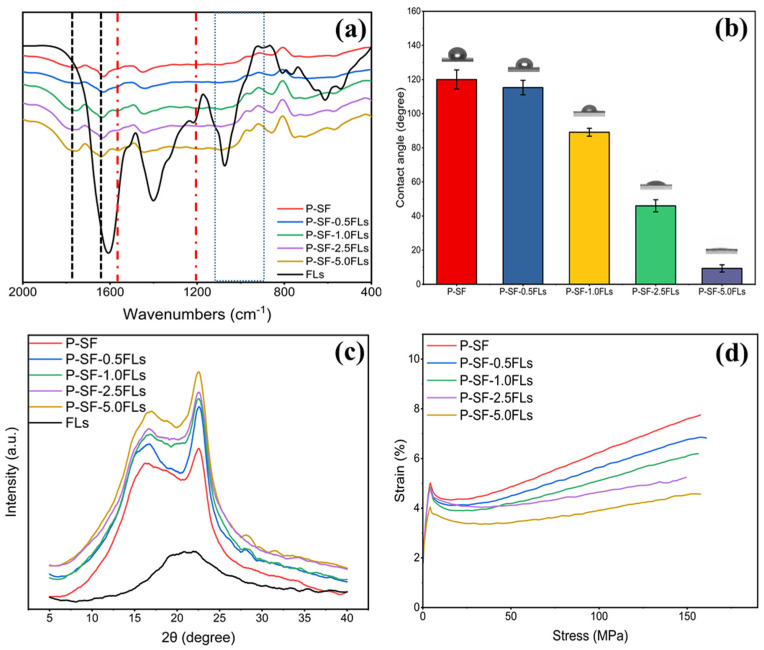
FTIR spectra (**a**), WCA (**b**), XRD spectra (**c**), and stress−strain curves (**d**) of different membranes loaded with FLs.

The physicochemical characterization of the FL-loaded nanofibre membranes is shown in Figure 3. The FTIR spectrum in Figure 3a indicates that the nanofibre membranes with different concentrations of FLs had characteristic peaks of PLGA and SF, and also contained characteristic peaks of FLs. This indicated that the addition of FLs did not affect the chemical structure of the blank control (P-SF). As shown in Figure 3b, the hydrophilicity of the P-SF nanofibre membrane increased with the addition of FLs. When the content of FLs increased from 0.5 to 5.0 mg/mL, the contact angle of the nanofibre membranes decreased from 118° to 17°. The WCA of the nanofibre membranes decreased with the addition of FLs, indicating a gradual increase in hydrophilicity with an increasing FL content. The FLs in green cocoons have some water solubility, which may be due to the hydroxy glycosidation of flavonoids or the polyhydroxyl group of flavonoid aglycone molecules increasing the water solubility [34,35]. Therefore, FLs have some water solubility, and the addition of FLs can enhance the hydrophilicity of the fibre membrane. XRD analysis showed that the characteristic peaks remained unchanged, suggesting that the addition of FLs had minimal impact on the secondary structure of the nanofibre films (Figure 3c). Figure 3d shows the effect of adding FLs on the mechanical properties of P-SF. The fracture strength of P-SF and P-SF-nFLs ranged from 7.7 ± 0.9 to 4.7 ± 0.1 MPa. The addition of FLs reduced the fracture strength of the nanofibre membranes, but had little effect on the elongation at break, which remained within the range required for skin wound healing [36]. Consequently, the incorporation of FLs into P-SF did not alter the nanofibre membrane’s structure and enhanced its hydrophilicity, thereby satisfying the mechanical requirements for skin wound healing.

### 2.3. Release of FLs from FL-Loaded P-SF Membranes

**Figure 4:**
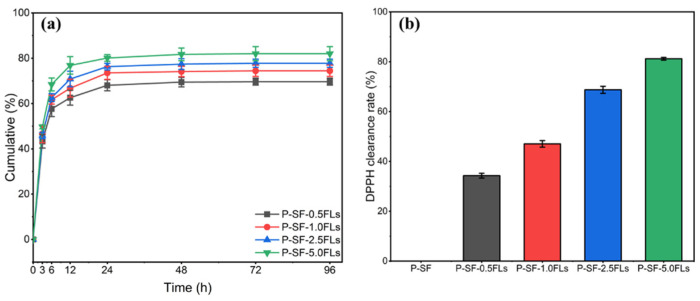
The sustained release (**a**) and DPPH clearing capacity (**b**) of P-SF-nFL membranes.

The encapsulation efficiency of P-SF-nFL membranes with different FL contents was investigated. The results showed that as the FL content increased from 0.5 to 5.0 mg/mL, the FL encapsulation efficiency of P-SF-nFLs membranes was 87.4 ± 2.1%, 89.1 ± 0.9%, 90.7 ± 1.3%, and 92.8 ± 1.7%. The results indicated a positive correlation between the encapsulation rate and the FL content in the membranes, which may be because the FLs were fully dispersed and encapsulated in the nanofibre membranes through solvent evaporation during electrospinning [37]. The application of FL-loaded nanofibre membranes in drug administration wound dressing is more beneficial when the encapsulation efficiency of FLs is high [38]. WCA results of P-SF-nFLs nanofibre membranes showed that loading FLs increased their hydrophilicity, indicating that the crude extract FLs from the green cocoon may have a certain water solubility due to the hydroxyl glycosylation of flavonoids or the increased water solubility of polyhydroxyl of flavonoid aglycone molecules. Moreover, PBS (pH = 7.4) was within the pH value range of human body fluids, so PBS (pH = 7.4) was selected as the sustained release medium to investigate the sustained release behavior of FL-loaded nanofibre membranes. Figure 4a presents the results of the investigation into the sustained release behaviour of the P-SF-nFL membranes. The FLs in the P-SF-nFL membranes exhibited a high sustained release rate from 0 to 12 h. After 72 h, the slow-release amount of FLs in P-SF-nFLs reached equilibrium in PBS. Furthermore, the amount of FLs released increased in direct proportion to the FL content. This was likely due to the higher encapsulation efficiency of the membranes with a higher FL content. The FL release rate was affected by the hydrophilicity and degradability of SF. In addition, a decrease in fibre diameter increased the specific surface area of the nanofibre membrane, which was conducive to a slow release and accumulation of FLs. At the same time, the results showed that FL-loaded nanofibre membranes exhibited good slow-release ability, which is beneficial for antibacterial and anti-inflammatory effects.

### 2.4. Anti-Oxidation and Antibacterial Properties of the P-SF-nFL Nanofibre Membranes

The antioxidant properties of FL-loaded nanofibre membranes were measured using the DPPH free radical scavenging method. The results, shown in Figure 4b, indicated that the slow-release solutions of other FL-loaded nanofibre membranes had stronger antioxidant effects compared with the blank group. Moreover, when the concentration of FLs was greater than 2.5 mg/mL, P-SF-2.5FLs and P-SF-5.0FLs exhibited DPPH free radical scavenging rates of 68.7% and 81.2%, respectively. Their clearance rate was higher than 50%, indicating that they had good antioxidant capacity. Other studies have also shown that hydroxylation modes of flavonoids, especially in the flavonoid structure of the B ring and the C2=C3 double bond conjugated with C4-carbonyl, methoxyl, and O3-H groups, can enhance antioxidant properties [39]. As a result, the antioxidant capacity of the nanofibre membrane was improved with an increase in FL content in the nanofibre membrane containing these structures.

**Figure 5:**
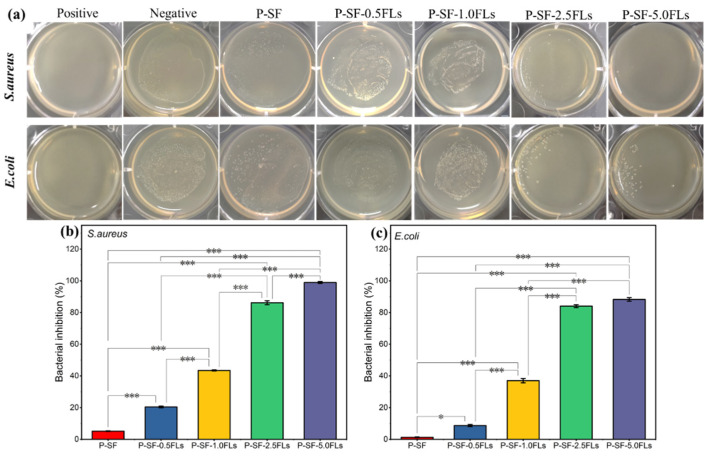
Antibacterial properties of different P-SF-nFL composite nanofibre membranes. Antibacterial effects (**a**) and the inhibition rates (**b**,**c**) of P-SF-nFL composite nanofibre membranes against *S. aureus* and *E. coli*. * *p* < 0.05; *** *p* < 0.001.

Wound healing and its associated bacterial infections are significant challenges in modern healthcare systems. During the initial stage of wound healing, some bacteria may breed on the wound surface, leading to wound infection and inflammation and thus hindering wound healing [40]. Therefore, wound dressings with antibacterial properties can reduce inflammation and promote wound healing. Flavonoids, particularly chalcone and certain flavonoid derivatives, have been reported to have high antimicrobial activity against multi-resistant Gram-negative and Gram-positive bacteria, including *E. coli* and *S. aureus* [41,42,43]. In this study, the antibacterial properties of FL-loaded nanofibre membranes against *S. aureus* and *E. coli* were investigated. As shown in Figure 5a, after co-culturing *S. aureus* and *E. coli* with different membranes for 18 h, the number of bacteria significantly decreased with an increased load of FLs, indicating that FL-loaded nanofibre membranes exerted an antibacterial effect on *S. aureus* and *E. coli*. The bacteriostatic rates (Figure 5b,c) indicated that when the dose of FLs was greater than 2.5 mg/mL, the bacteriostatic rates of the nanofibre membranes against both bacteria were greater than 80%. The growth inhibition rates of *S. aureus* and *E. coli* were 98.9% and 88.3%, respectively, when P-SF was loaded with 5.0 mg/mL FLs. The results showed that the bacteriostatic activity against *S. aureus* and *E. coli* gradually increased with an increase in FLs. This indicated that the antimicrobial properties of FL-loaded nanofibre films are dependent on the dose of FLs. The results also showed that P-SF-nFLs had a stronger effect on Gram-positive bacteria than on Gram-negative bacteria. The main reason may be that FLs comprise a mixture, and some flavonoids contained in FLs have stronger antibacterial properties against Gram-positive bacteria than Gram-negative bacteria [24,41]. Therefore, the membranes showed a good antibacterial effect and can be used as a dressing for wound healing.

### 2.5. Biocompatibility of the P-SF-nFLs Nanofibre Membranes

**Figure 6:**
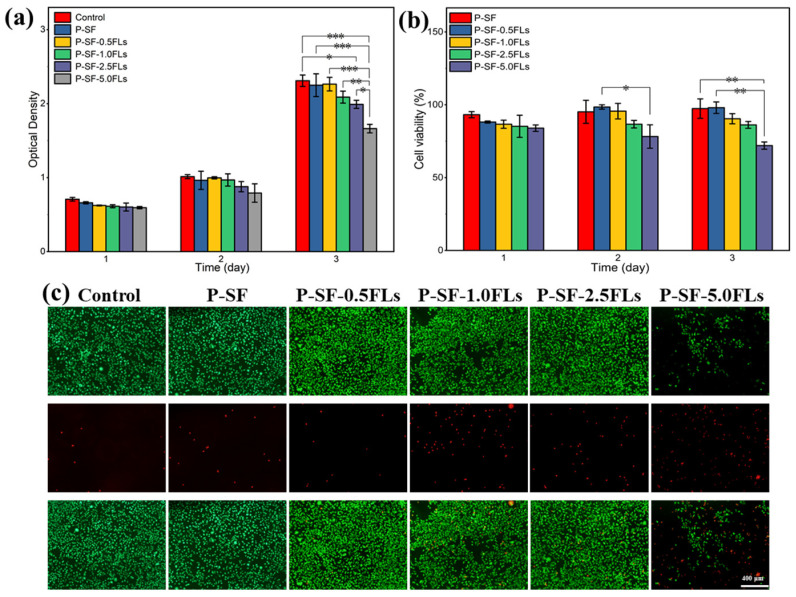
Cytotoxicity of different P-SF-nFL nanofibre membranes on normal L929 cells as determined by an MTS assay (**a**), cell survival rate of L929 cells (**b**), live and dead cell staining of L929 cells on the third day of incubation (**c**). * *p* < 0.05; ** *p* < 0.01; *** *p* < 0.001, the scale bar represents 400 μm.

The P-SF-nFLs nanofibre membranes used as wound dressings must have good biocompatibility. To assess cell compatibility, L929 cells were co-cultured with the composite membranes, and the results were analysed using MTS and live/dead cell staining, as shown in Figure 6. L929 cells were grown on each nanofibre membrane for 3 days, and the cell viability on all membranes was above 80%, indicating good cell compatibility, similar to the control group, except for P-SF-5.0FLs (Figure 6a,b). The cell status was also studied using live/dead cell staining, and the results are presented in Figure 6c. The L929 cells were able to attach to the membrane surface and grow due to the porous structure and high specific surface area provided by the electrospun membrane, which was conducive to cell adsorption and growth. With the exception of P-SF-5.0FLs, most cells in the other groups were viable (green), while the number of dead cells (red) increased gradually with higher concentrations of FLs. Therefore, the FLs released from the P-SF membranes loaded with low levels of FLs did not affect cell growth or proliferation, which was consistent with the MTS results. In summary, these results demonstrated that the nanofibre membrane loaded with FLs at a concentration of less than 5 mg/mL exhibited good biocompatibility. Therefore, the P-SF-2.5FL nanofibre membrane not only met the cell compatibility conditions, but also had good antibacterial properties, which can be used for follow-up experiments.

### 2.6. Anti-Inflammatory Analysis of P-SF-nFLs Nanofibre Membranes

**Figure 7:**
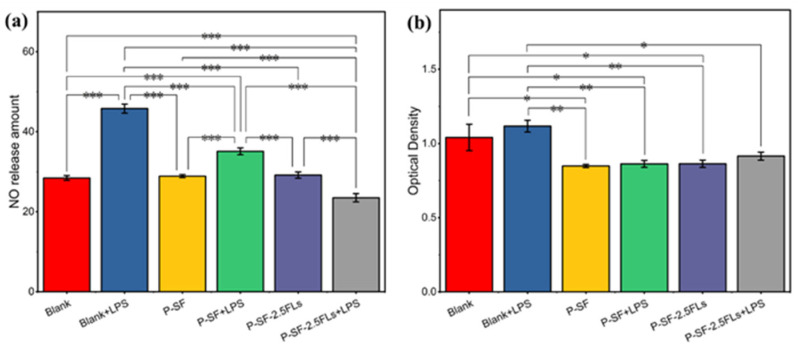
(**a**) Inhibition of P-SF and P-SF-2.5FLs on NO production in LPS-stimulated RAW264.7 cells; (**b**) cytotoxicity of P-SF and P-SF-2.5FLs on RAW264.7 cells as measured by an MTS assay. * *p* < 0.05; ** *p* < 0.01; *** *p* < 0.001.

The process of reconstructing wounded skin is accompanied by a period of an inflammatory response; therefore, wound dressings possess anti-inflammatory properties that facilitate wound healing and reduce the formation of scar tissue. Inflammation can be induced by nitric oxide synthase, resulting in the production of NO. To characterize anti-inflammatory activity, the amount of NO release was measured in cells induced by LPS. Figure 7a shows that the P-SF nanofibre membrane loaded with FLs significantly reduced the release of NO in LPS-induced cells compared with the other induced and non-induced groups. P-SF-2.5FLs released FLs, which reduced the release of the inflammatory factor TNF-α and inflammatory mediator NO by inhibiting the transcriptional signalling pathway of macrophages [25,43,44]. This suggests that P-SF-2.5FLs can effectively inhibit the inflammatory response. Figure 7b illustrates that the cell survival rate of the P-SF-2.5FL group induced by LPS was 83%, indicating that its inhibitory effect on the inflammatory response was not toxic to cells. The cell viability of the other groups was also above 80%, demonstrating that P-SF and P-SF-2.5FLs were biocompatible with mouse macrophage RAW264.7 cells.

### 2.7. In Vivo Wound Healing and Histological Analysis

**Figure 8:**
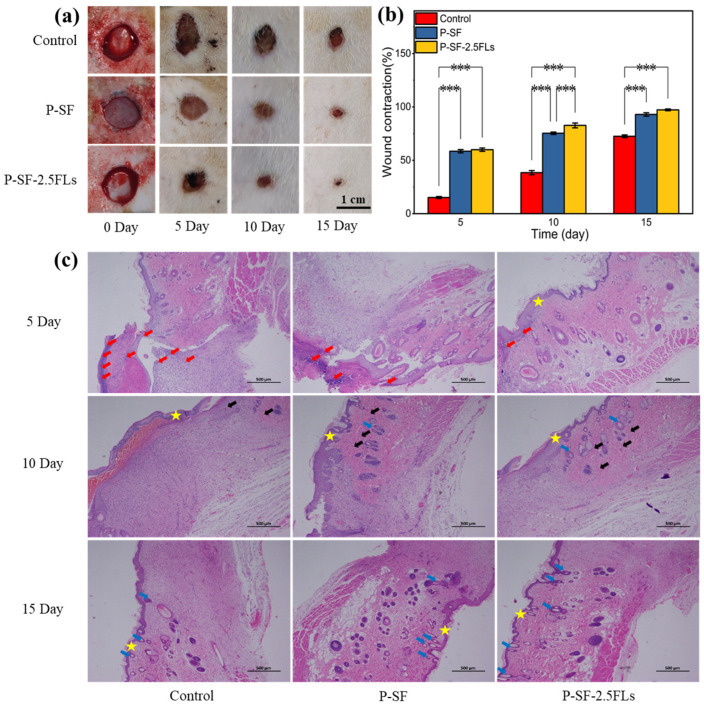
(**a**) Photographs of wounds treated with dressings; (**b**) assessment of the wound size reduction; and (**c**) H&E histological analysis of wounded rat skin on days 5, 10, and 15. Red arrows: inflammatory cells; black arrows: capillaries; blue arrows: hair follicle structure; star: skin. *** *p* < 0.001.

The above studies showed that although P-SF-5.0FLs had good antibacterial properties, their cell survival rate was less than 80%. In contrast, P-SF-2.5FL nanofibre membranes exhibited notable slow-release effects, commendable antibacterial properties, anti-inflammatory activity, and cytocompatibility. To further evaluate the wound healing effects of P-SF-2.5FL membranes in vivo, a 1 cm full-layer skin wound was made on the back of SD rats as a model for a skin wound. Figure 8a shows the wound appearance of SD rats at different time points after full skin resection. On the fifth day, the wounds of the three groups of rats began to scab, and the wound area of each group was reduced, as determined by a statistical analysis. As shown in Figure 8b, on the 10th day, shrinkage rates for the blank, P-SF, and P-SF-2.5FL groups were 38.4%, 75.3%, and 82.7%, respectively. The healing effect of the P-SF-2.5FL group was better than that of the P-SF and control groups. On the 15th day, the wounds of all groups were nearly healed. The group treated with P-SF-2.5FLs demonstrated earlier scab shedding compared with the other two groups, indicating superior epithelialization and wound healing. In addition, 92.9% and 97.3% wound shrinkage were observed in the P-SF and P-SF-2.5FLs groups, respectively, while the control group was 72.5% (Figure 8b). These results indicated that P-SF-2.5FLs had better wound healing effect than the other groups. This was mainly because the structure of the nanofibre membrane effectively absorbed exudate and the addition of FLs provided excellent antibacterial and anti-inflammatory properties. FL-loaded nanofibre membranes can help prevent infection and pus formation during the wound healing process, and reduce the duration of the inflammatory period.

H&E staining further verified the wound healing process, and the results are shown in Figure 8c. On the fifth day after the operation, it was observed that a large number of inflammatory cells infiltrated the wound in the blank control group, causing a strong microbial-mediated acute immune inflammatory reaction that delayed the process of wound healing. By contrast, the nanofibre membrane induced a relatively mild inflammatory response with few inflammatory cells, which promoted wound healing, especially in the P-SF-2.5FL group, where even fewer inflammatory cells were observed. On the 10th day, the P-SF-2.5FL group showed the formation of new granulation tissue, capillaries, and a small number of hair follicle structures. The findings indicated that the introduction of FLs to P-SF-2.5FLs accelerated the rapid healing of wounds, which was attributed to the antimicrobial, anti-inflammatory, and antioxidant properties of FLs. These results were consistent with the previously conducted in vitro tests of P-SF-2.5FLs. It is noteworthy that P-SF-2.5FLs promoted the repair of damaged skin, and a large number of hair follicle structures were observed in skin tissue, while the control group had fewer hair follicles. In conclusion, it can be stated that P-SF-2.5FLs promoted wound healing due to its antibacterial, anti-inflammatory, and antioxidant effects.

### 2.8. Effect of FLs on Expression of Genes Involved in Wound Healing

**Figure 9:**
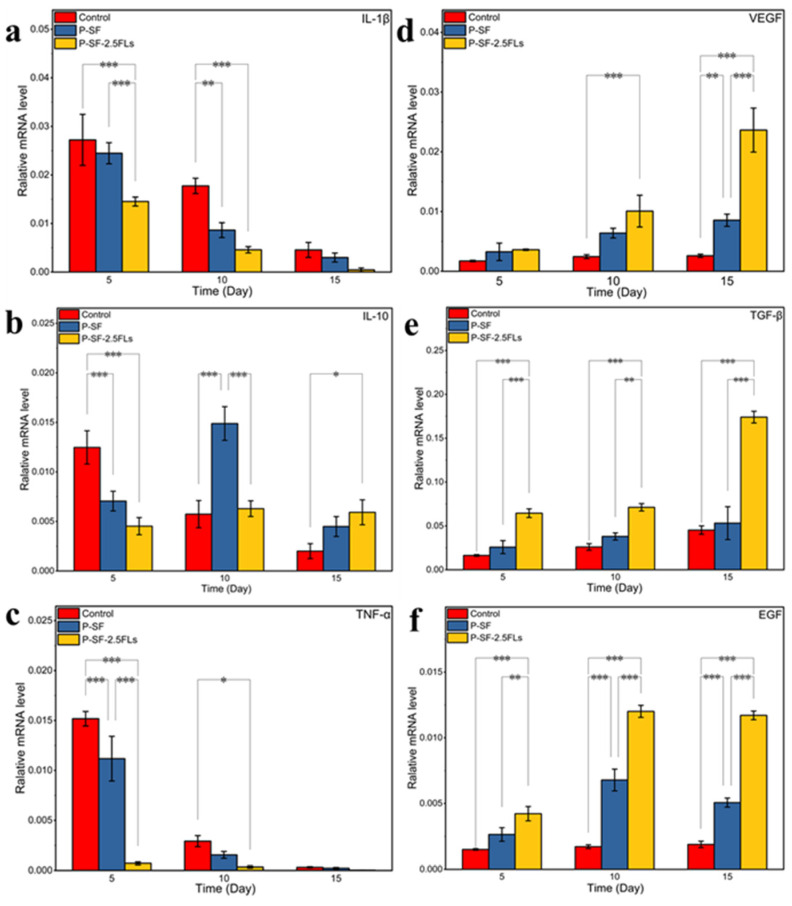
Expression levels of relevant inflammatory factors (**a**–**c**: IL-1β, IL-10, and TNF-α) and growth factors (**d**–**f**: VEGF, TGF-β, and EGF) in wound regeneration under different treatments. * *p* < 0.05; ** *p* < 0.01; *** *p* < 0.001.

To investigate the molecular mechanism by which the nanofibre membranes promoted wound healing, the expression levels of three growth factors (VEGF, TGF-β, and EGF) and three inflammatory factors (IL-1β, TNF-α, and IL-10) were determined in rat regenerated skin, as shown in Figure 9. IL-1β promotes inflammatory responses and can stimulate the production of related inflammatory transmitters and other cytokines that cause tissue damage [45]. TNF-α is the earliest inflammatory mediator in the inflammatory response, regulating metabolic activity in other tissues and promoting the synthesis and release of other cytokines [46]. The levels of IL-1β and TNF-α were high at the start of the inflammatory response and gradually decreased towards the end of the response period (Figure 9a,c). Compared with the control group, the expressions of IL-1β and TNF-α in the P-SF-2.5FLs group were significantly reduced on days 5 and 10. With the progression of wound healing, the expression of IL-10 in the P-SF-2.5FL group first increased and then decreased (Figure 9b). During the late inflammatory response, high expression of IL-10 may contribute to the end of the inflammatory response period and subsequent recovery. The results demonstrated that the FLs released from the P-SF-2.5FL nanofibre membrane exerted anti-inflammatory activity mainly by downregulating the expression of pro-inflammatory cytokines such as IL-1β and TNF-α. Therefore, the P-SF-2.5FLs nanofibre membrane effectively promoted wound healing.

During the wound healing process, growth factors such as VEGF, TGF-β, and EGF play crucial roles. VEGF promotes angiogenesis and vascular endothelial cell proliferation, while TGF-β promotes fibroblast growth and collagen expression, and inhibits extracellular matrix degradation [47]. As shown in Figure 9d,e, the expression levels of VEGF and TGF-β increased on the 10th and 15th days, and there were significant differences. The expression levels of the P-SF-2.5FLs group were the highest among them. This was mainly due to vascular regeneration of the regenerated skin on the 10th and 15th days, and an increase in tissue remodelling activity. This indicated that P-SF-2.5FLs had a promoting effect on the expression of VEGF and TGF-β. In addition, EGF can promote the repair of proliferative epidermal cells and accelerate the growth of skin and mucosa [48]. P-SF-2.5FLs also promoted EGF expression, as shown in Figure 9f. On day 10, following the end of the inflammatory response period, the expression of EGF significantly increased in the P-SF-2.5FLs group. At this point, granulation tissue may have fully formed, and the synthesis of various nutrients needed for wound healing has begun. EGF expression was reduced on day 15, possibly indicating that the repair process was nearing completion. In conclusion, P-SF-2.5FLs may enhance the expression of growth factors and regulate the expression of inflammatory factors during different stages of wound healing, thereby promoting wound healing.

## 3. Materials and Methods

### 3.1. Materials

Green cocoons were produced by feeding silkworms. Poly (lactide-co-glycolide acid) (PLGA, 50/50, M_W_: 100,000–120,000) was purchased from Jinan Daigang Biotechnology Co., Ltd. Hexafluoroisopropanol (HFIP) (purity > 99.5%) was obtained from Aladdin Chemical Co. Ltd. (Shanghai, China). 2,2-Diphenyl-1-picrylhydrazyl (DPPH) was purchased from Shanghai Macklin Biochemical Co., Ltd. Dulbecco’s modified Eagle medium (DMEM), fetal bovine serum (FBS), trypsin-EDTA, and penicillin-streptomycin were purchased from Gibco BRL, Rockville, MD, USA. An MTS cell proliferation and cytotoxicity assay kit was acquired from Promega, Madison, WI, USA. A viability/cytotoxicity assay kit for animal live/dead cells was obtained from US Everbright Inc. Other chemicals were purchased from Chongqing Taixin Chemical Co. (Chongqing, China).

### 3.2. Extraction of Flavonoids and Preparation of Regenerated Silk Fibroin

FLs were extracted from the green cocoons (G200) using 40% ethanol (solid–liquid ratio 1:20) with ultrasonic assistance for 20 min. The process was repeated twice, and the supernatant was collected by centrifugation at 8000× *g* for 5 min. The concentrated solution was obtained by removing the ethanol through vacuum rotary evaporation. The solution was then used to prepare crude flavonoid extract via freeze-drying. After extracting the flavonoids, the cocoons were dried at 45 °C to prepare the regenerated SF using previously studied methods [49].

### 3.3. Fabrication of PLGA/SF-nFL Nanofibre Membranes

PLGA, SF, and different concentrations of FLs (0.5, 1.0, 2.5, 5.0 mg/mL) were added to the HFIP solution according to the proportions given in Table 1, with a total weight of 20 g. After stirring and dissolving for 6 h, the solution was transferred to a syringe for spinning using an electrostatic spinning machine (TL-Pro-BM, Tongli Micro, Shanghai, China). The electrospinning parameters were slightly modified based on our previous study, in which a high voltage of −1 to 17 kV was applied between the needle and the collector at a distance of 12 cm [16]. Subsequently, the nanofibre membranes were obtained by removing the tinfoil following vacuum drying.

### 3.4. Characterization of Composite Nanofibre Membranes

The morphologies of the nanofibre films were observed by scanning electron microscope (SEM, SU8020, Tokyo, Japan) under a high vacuum acceleration voltage of 5–20 kV after gold spraying. Fibre diameters were measured using ImageJ software and then statistically analysed. The composition of the nanofibre membranes was examined using Fourier transmission infrared (FTIR, Bruker, Karlsruhe, Germany) spectroscopy in a scanning range of 4000–400 cm^−1^. The water contact angle (WCA) of the nanofibre membranes (20 mm × 10 mm) was measured using a contact angle meter (OCA15EC, Datephysics, Filderstadt, Germany) at 25 °C (n = 3) to evaluate their hydrophilicity. The mechanical properties of the nanofibre membranes (30 mm × 10 mm) were tested at 25 °C with a tensile speed of 5 mm/min using a universal material testing machine (Autograph AGS-X, Kyoto, Japan). The crystal structure of the prepared samples was analysed by an X’Pert PRO MPD X-ray diffractometer (XRD, Panalytical, Almelo, Holland) with Bragg angles (2θ) ranging from 5° to 40°.

### 3.5. In Vitro Release of FLs from FL-Loaded Membranes

The nanofibre membranes loaded with different contents of FLs were weighed and dissolved in HFIP. After centrifuging the mixture, the upper layer was collected for subsequent analysis of the encapsulation efficiency of FLs. The sheared nanofibrous membrane samples were placed in test tubes containing PBS (pH = 7.4) and incubated in an incubator at 37 °C with shaking at 150 rpm/min. At specified time points, each sample was transferred to a new tube containing fresh PBS for analysing the sustained release. The release from each stage was collected by periodically repeating this operation. The content of FLs was determined at 510 nm using a BioTek microplate reader (VT, USA), following the method of Lu et al. [50]. The encapsulation efficiency (EE) and sustained release efficiency (SE) of the FLs were calculated by the following formulas: EE (%) = (amount of FLs in the membrane/total amount of FLs) × 100%; SE (%) = (F_t_/F) × 100%, where F_t_ and F represent the mass of the FLs released at time t and the total FL quantity, respectively.

### 3.6. Antioxidant and Antimicrobial Activity of the Nanofibre Membranes

The FLs of the FL-loaded nanofibre membranes were released for 72 h according to the method described in Section 3.5 to obtain an FL-containing sustained release solution. Then, 0.5 mL of the sustained release solution was added to 1 mL of a 0.15 mM DPPH solution as the experimental group (*A*_0_), absolute ethanol as the control group (*A*_1_), and 0.15 mM DPPH as the negative control group (*A*_2_), and absorbance was measured at 517 nm. The DPPH clearance rate (*R*) was calculated as follows (1) [51]:

(1) $$R\left(\%\right)=(1-(A_{0}-A_{1})/A_{2})\times 100\%$$

The antibacterial properties of the FL-loaded nanofibre membranes were evaluated using our previous research method to test the antibacterial effect against *Escherichia coli* (*E. coli*) and *Staphylococcus aureus* (*S. aureus*) [49]. First, a standard bacterial solution was prepared by a four-step dilution method, resulting in concentrations of 1.5 × 10^5^–3.5 × 10^5^ CFU/mL. To a 15 mL centrifuge tube, 0.05 g of a UV-sterilized sample, 7 mL of PBS, and 0.5 mL of the standard bacterial solution were added. The mixture was then incubated at 37 °C for 18 h. The negative control for the bacterial culture was conducted without samples, while the positive control was conducted with 25 μL of gentamicin sulfate. Bacterial dilutions co-cultured with the samples were spread on a solid medium and incubated at 37 °C for 18 h. The number of living bacteria was then counted and the antibacterial rate (*W*) was calculated according to Equation (2):

(2) $$W(\%)=\frac{C-E}{C}\times 100\%$$

where *C* and *E* represent the number of bacteria in the negative control group and experimental group, respectively.

### 3.7. In Vitro Biocompatibility of PLGA/SF-nFL Membranes

The cytocompatibility of the nanofibre membranes loaded with FLs was evaluated by means of MTS and cell staining assays. Nanofibre membranes 8 mm in diameter were placed at the bottom of each well of a 48-well plate and sterilised by UV irradiation. L929 cells (1 × 10^4^ cells/mL) were then inoculated into the 48-well plates, while the controls did not include the nanofibre samples. The cells were then cultured for 3 days at 37 °C in a cell incubator. The viability of the L929 cells was assessed by an MTS assay at 1, 2, and 3 d and calculated using the following formula: Cell viability (%) = (the optical density of each sample group/the optical density of control group) × 100%. After 3 d, L929 cells were fluorescently stained, and the dead or live status of the cells was observed by fluorescence microscopy.

### 3.8. Anti-Inflammatory Testing of PLGA/SF-nFL Membranes

An inflammatory model was established by inducing mouse macrophage RAW264.7 cells with lipopolysaccharide (LPS), and the anti-inflammatory properties of FL-loaded nanofibre membranes were evaluated by a NO detection kit. The membranes were prepared as described in Section 3.7. They were then placed in a 48-well plate and each well was seeded with RAW 264.7 cells (1 × 10^4^/mL, 60 µL) and supplemented with 540 µL of culture medium or culture medium containing 0.5 µg/mL LPS. Cells were incubated in a 5% CO_2_ incubator at 37 °C for 24 h. After 24 h, a NO detection kit reagent was used to react with NO released from the supernatant and was measured at 550 nm using a BioTek microplate reader (USA). Blank controls were used for cells cultured in wells without samples. The viability of LPS-induced mouse macrophage RAW264.7 cells was determined using the MTS method.

### 3.9. In Vivo Wound Healing with Nanofibre Membranes

To assess wound healing in vivo, a full-thickness wound defect model in a rat dorsum was established. All experiments with rats were conducted in accordance with the National Institutes of Health Guide for the Care and Use of Laboratory Animals (NIH Publication No. 8023, revised 1978) and in accordance with the Guide for the Care and Use of Laboratories, College of Animal Science and Technology, Southwestern University. The methodology for animal experiments was modified slightly from the previous version [16]. The Sprague Dawley (SD) rats were randomly divided into three groups (n = 3/group) based on three stages (5, 10, and 15 days). After being anaesthetized, the SD rats were depilated and sterilized with 75% alcohol. Then, four full-thickness skin wounds, each with a diameter of 1 cm, were created bilaterally on the back of each rat. The wounds were spaced 2 cm apart, with two wounds on each side. P-SF and P-SF-2.5FL nanofibre membranes were applied to the wounds as the experimental group. Unfortunately, no samples were available for the control group, and the nanofibre membranes were wrapped around the wounds with a bandage. Observations of the wounds were recorded every 5 days, and the size of the wounds was measured using ImageJ software. Wound shrinkage (*W*) was calculated by Equation (3):

(3) $$W\;(\%)=\frac{S_{0}-S_{n}}{S_{0}}\times 100\%$$

where *S*_0_ and *S_n_* represent the wound areas on days 0, 5, 10, and 15.

### 3.10. Histological Analysis

The wound and surrounding skin samples were collected on days 5, 10, and 15 and then fixed in 4% paraformaldehyde, embedded in paraffin, and cut into vertical sections of 4 μm thickness. The sections were stained with haematoxylin–eosin (H&E), and the histopathological changes of the healed wounds were observed and analysed under a microscope.

### 3.11. Real-Time qPCR Examination

Real-time quantitative polymerase chain reaction (RT-qPCR) was used to study the expression of genes related to wound healing. All samples collected on days 5, 10, and 15 were ground using liquid nitrogen and total RNA was extracted with Trizol (Invitrogen, Carlsbad, CA, USA). The RNA was then reverse-transcribed using a kit from TAKARA (China). The RT-qPCR used a SYBRE Green premix PRO Taq HS qPCR kit (Yeasen, Hunan, China) and was carried out by BX53 (Olympus, Tokyo, Japan). Six genes, interleukin-1β (IL-1β), tumour necrosis factor-α (TNF-α), interleukin-10 (IL-10), vascular endothelial growth factor (VEGF), transforming growth factor-β (TGF-β), and epidermal growth factor (EGF) were analysed for expression levels with GAPDH as a reference gene. Relative gene expression was measured by the 2^−ΔΔCT^ method. The sequences of relevant primers are listed in Table 2.

### 3.12. Statistical Analysis

All experimental data are expressed as mean ± standard deviation. All analyses of variance are performed to test the significance of the results by *t*-test. A *p*-value < 0.05 was denoted as statistically significant; * is *p* < 0.05, ** is *p* < 0.01, and *** is *p* < 0.001.

## 4. Conclusions

In this study, a P-SF nanofibre membrane loaded with FLs was prepared using green cocoons as regenerated silk fibroin and FL material by blending electrospinning. The prepared films had a good slow-release effect, and the cumulative release of FLs was up to 82% after 72 h due to the SF blending. By releasing FLs, the P-SF-2.5FLs nanofibre membrane demonstrated effective antioxidant, antibacterial, and anti-inflammatory properties while maintaining ideal biocompatibility. Further evaluation of wound healing in animal models demonstrated that the P-SF-2.5FLs nanofibre membrane exhibited anti-inflammatory activity, the mechanism of which may be the downregulation of pro-inflammatory cytokine expression during the inflammatory phase, thereby promoting wound contraction and healing. In summary, the P-SF-2.5FL nanofibre membrane prepared in this study exhibited good antibacterial properties, anti-inflammatory effects, and biocompatibility, and provides a new approach for treating skin wounds. The use of green cocoons in wound healing overcomes the limitations of material development and taps into the potential of natural green cocoons.

## Figures and Tables

**Table 1:** The parameters of the composite nanofibre membranes.

Sample Name	Concentration of PLGA (wt%)	Concentration of SF (wt%)	Concentration of FLs (mg/g to Dry Matter)
SF	-	10	-
50PLGA/50SF	5	5	-
60PLGA/40SF	6	4	-
80PLGA/20SF	8	2	-
PLGA	10	-	-
80PLGA/20SF/nFLs	8	2	3, 6, 15, 30

**Table 2:** The primer sequences of related genes.

Gene	Forward Primer	Reverse Primer
GAPDH	CCTTCCGTGTTCCTACCCC	ACCAGGAAATGAGCTTGACA
IL-1β	AACAAACCCTGCAGTGGT TCG	AGCTGCTTCAGACACTTGCAC
IL-10	CCATCATGCCTGGCT CAG CAC	TGTACTGGCCCCTGCTGATCC
TNF-α	GAAGCTCCCTCAGCGAGGACA	TTGGGCCAGTGAGTGAAAGGG
VEGF	GAGACCCTGGTGGACATCTT	GATCCGCATGATCTGCATAGG
TGF-β	AACAATTCCTGGCGTTACCTT	TGTATTCCGTCTCCTTGGTTC
EGF	GCCACGGTTACATTCACTCC	CCAAATCGCCTTCTCTTTCA

## Data Availability

The data presented in this study are available on request from the corresponding author.

## Supplementary Materials

The following supporting information can be downloaded at: https://www.mdpi.com/article/10.3390/ijms25179263/s1.
