# Supplementary material for: A PLGA/Silk Fibroin Nanofibre Membrane Loaded with Natural Flavonoid Compounds Extracted from Green Cocoons for Wound Healing
Source: Int J Mol Sci. 2024 Aug 27;25(17):9263. doi: 10.3390/ijms25179263 (PMC11394843; doi:10.3390/ijms25179263)
Supplement: Supplementary file 1 [file ijms-25-09263-s001.zip › ijms-3146822-supplementary.pdf]

A PLGA/Silk Fibroin Nanofibre Membrane Loaded with Natural Flavonoid Compounds Extracted from Green Cocoons for Wound Healing

Xiang Chen [1,2], Jiaqi Liu [1,2], Yaru Lu [1], Huijun Liu [1,2], Lan Cheng [1,2], Zhi Li [1,2], and Fangyin Dai [1,2,3,*]

[1] State Key Laboratory of Resource Insects, Institute of Sericulture and Systems Biology, Southwest University, Chongqing, 400715, China; chenxiang262@163.com (X.C.)

[2] Chongqing Engineering Research Center of Biomaterial Fiber and Modern Textile, College of Sericulture, Textile and Biomass Science, Southwest University, Chongqing 400715, China

[3] Key Laboratory of Sericulture Biology and Genetic Breeding, Ministry of Agriculture and Rural Affairs, College of Sericulture, Textile and Biomass Sciences, Southwest University, Chongqing, 400715, China

* Correspondence: fydai@swu.edu.cn

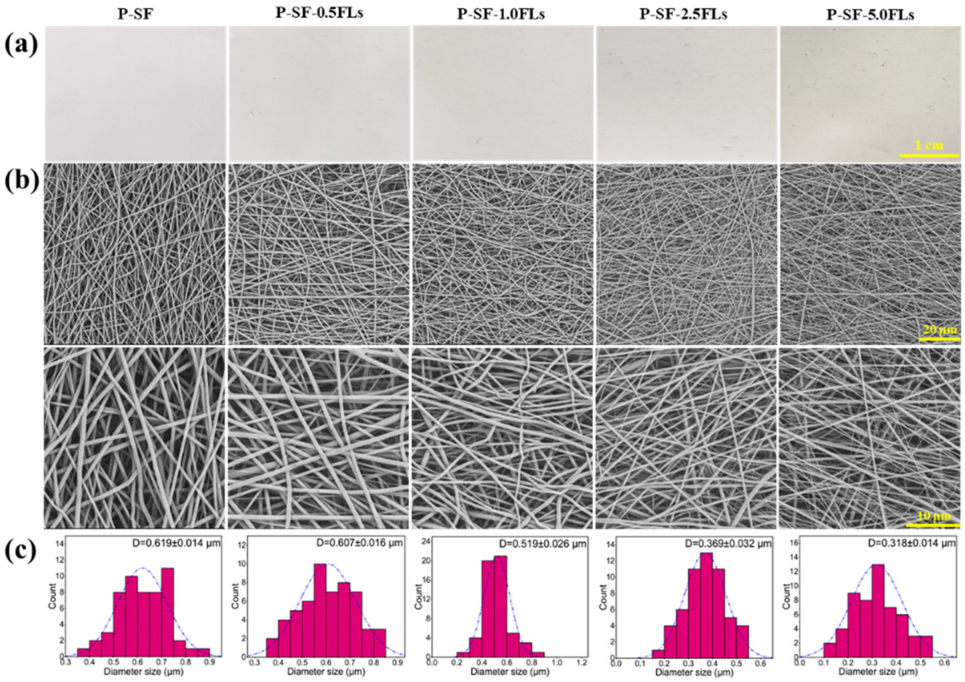

Figure S1. The optical photographs (a), SEM images (b), and the fibre diameter distributions (c) of the composite membranes loaded with FLs.
